# Correlation of Morphological Findings of Endometrium With Concerned Hormone Levels in Patients With Abnormal Uterine Bleeding: A Narrative Review

**DOI:** 10.7759/cureus.30063

**Published:** 2022-10-08

**Authors:** Tanvi T Bhardwaj, K.M. Hiwale, Sunita Vagha

**Affiliations:** 1 Pathology, Jawaharlal Nehru Medical College, Datta Meghe Institute of Medical Sciences, Wardha, IND

**Keywords:** menstrual, hormone, morphology, endometrium, aub

## Abstract

Abnormal uterine bleeding (AUB) is a distressing menstrual condition that continues to be one of the most common rationales for hysterectomy in underdeveloped countries. It can be either focal (breakthrough bleeding) or diffuse (bleeding throughout the body) (withdrawal bleeding). AUB has a negative impact on a female's physical, social, and emotional well-being. It is caused by chronic endometritis, micro erosions, or vascular fragility caused by micro-vessel structural anomalies. Polyps, submucosal leiomyoma, atrophy, and cancer can all produce endometritis and micro erosions in otherwise healthy endometrium (organic causes). In hyperplasia and endometrial cancers, especially type I, estrogen and progesterone hormones are also expressed. The level of these hormones gives prognostic information. They also provide amenability to hormonal therapy. Hormonal imbalance is the main component involved in the pathogenesis of dysfunctional uterine bleeding (DUB).

## Introduction and background

One of the most prevalent reasons that women visit their gynecologists is for abnormal uterine bleeding (AUB). AUB is clarified as any deviation from the normal menstrual cycle, including changes in regularity, monthly frequency, flow duration, and blood loss. Further subcategories of AUB can be found based on the amount of menstruation, regularity, frequency, term, chronicity, and timing of reproductive status.

**Figure 1:**
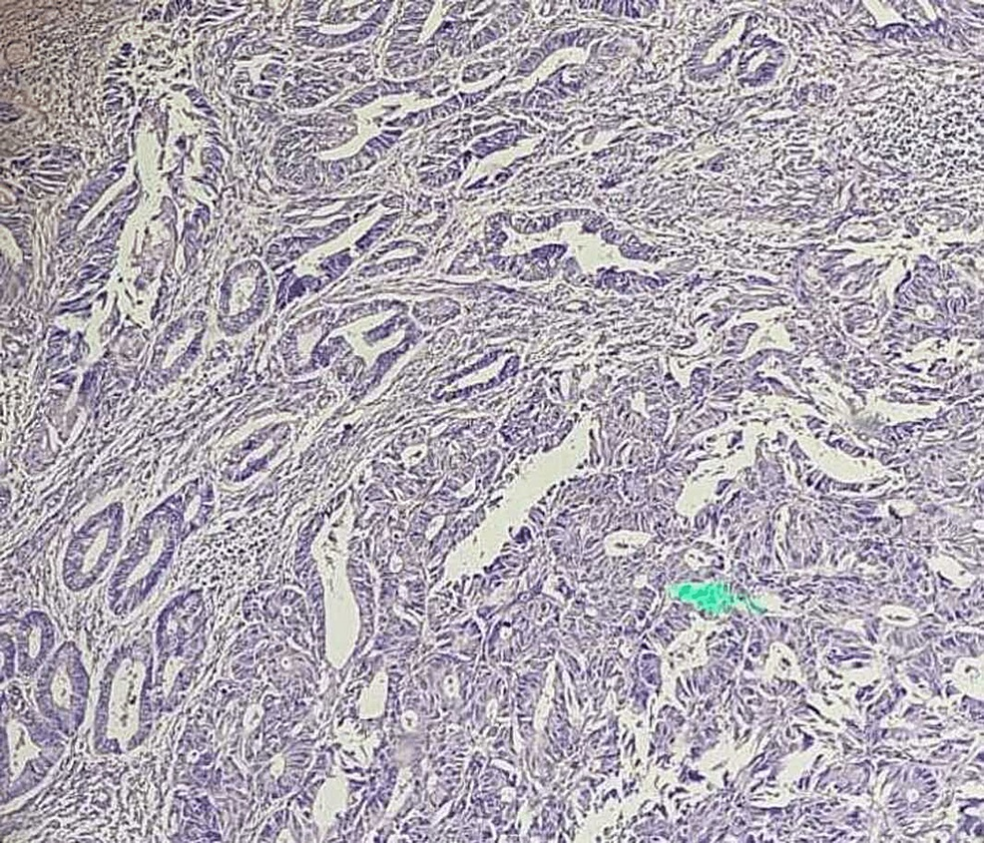
Endometrial carcinoma (H and E stain) H and E = hematoxylin and eosin

AUB can strike anyone at any age and manifest itself in a variety of ways. Women of reproductive age may experience abnormal uterine bleeding for a variety of reasons, from physiological processes to malignant tumors. These reasons include organic, systemic, and hormonal reactions. Fibromyoma, adenomyosis, endometrial polyps, ovarian tumors, pelvic inflammatory disease (PID), endometrial hyperplasia, endometrial carcinoma (Figure 1), hormonal imbalances (such as hypothyroidism), and hypothalamic-pituitary disorders are all possible reasons. AUB occurs in a high percentage of patients without any systemic reasons or organic genital tract diseases, and this is referred to as dysfunctional uterine bleeding (DUB).

AUB has a negative impact on a woman's physical, social, and emotional well-being. Women with excessive bleeding of undetermined onset have been seen to withdraw from typical activities because they may require constant access to pads and/or tampons, and they fear social activity or sexual connections because they believe they are approaching a heavy period. In many situations, the symptoms are modest and are caused by self-limiting changes in normal physiology [1].

Atrophic endometrium, endometritis, endometrial polyps, endometrial hyperplasia, and endometrial carcinoma are a few of the pathologies that can be detected histologically in AUB. Other pathologies include luteal phase defect, pill effect, and hormonal imbalance pattern (disorderly proliferative endometrium, non-secretory endometrium with stromal and glandular breakdown). However, endometrial abnormalities were seen in only about half of the cases of AUB, and the clinical picture was dominated by a hormone imbalance pattern [2-3].

In hyperplasia and endometrial cancers, especially type I, estrogen and progesterone hormones are also expressed. The level of these hormones gives a constituent of prognostic information. Additionally, they offer the potential for hormonal therapy. Hormonal imbalance is the leading component involved in the pathogenesis of DUB. This imbalance is better analyzed by histopathological evaluation of the endometrium.

## Review

Causes of AUB

AUB can be caused by systemic or functional causes. Metrorrhagia, menorrhagia, intermenstrual bleeding, and polymenorrhea are all potential symptoms. Histological changes of the endometrium can be used to determine the underlying pathology, taking into account age, menstrual cycle phase, and the usage of any exogenous hormones. Further causes include psychological stress, weight (obesity, anorexia, or a quick shift), exercise, endocrinopathies, neoplasms, medications, or unknown etiologies, according to research. Patients with abnormal uterine bleeding are more likely to be in the age category of 40 to 49 years, with a frequency of around 64% in the population pool [4].

Diagnosis

DUB is a subtype of AUB that includes non-organic origin of abnormal bleeding. It's found in half of all the females under the study with AUB. When there is AUB, an endometrial biopsy is usually recommended to rule out any organic pathology. Age and menstrual history are particularly significant factors because the causes of AUB vary with age and menstrual state. Women of reproductive age frequently experience pregnancy issues, including termination, whereas postmenopausal women are more likely to experience atrophy and organic diseases [4].

**Figure 2:**
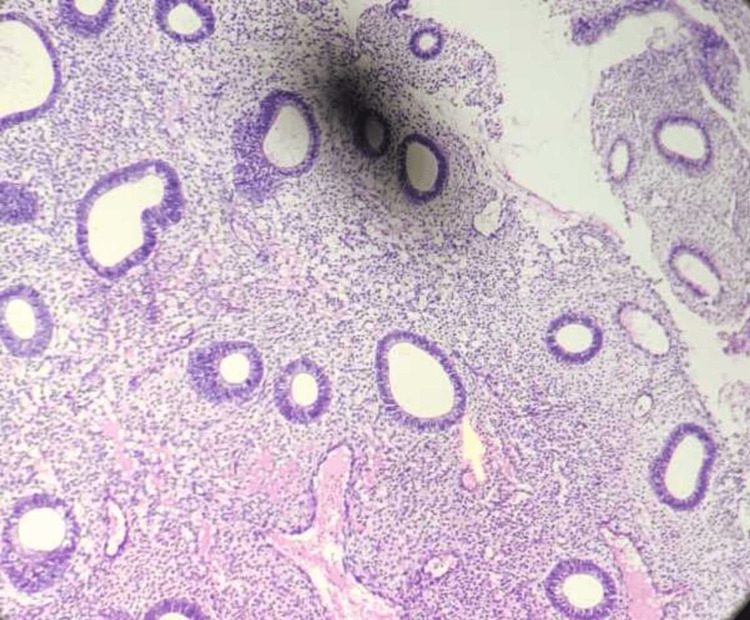
Endometrium showing proliferative phase (H and E stain) H and E = hematoxylin and eosin

**Figure 3:**
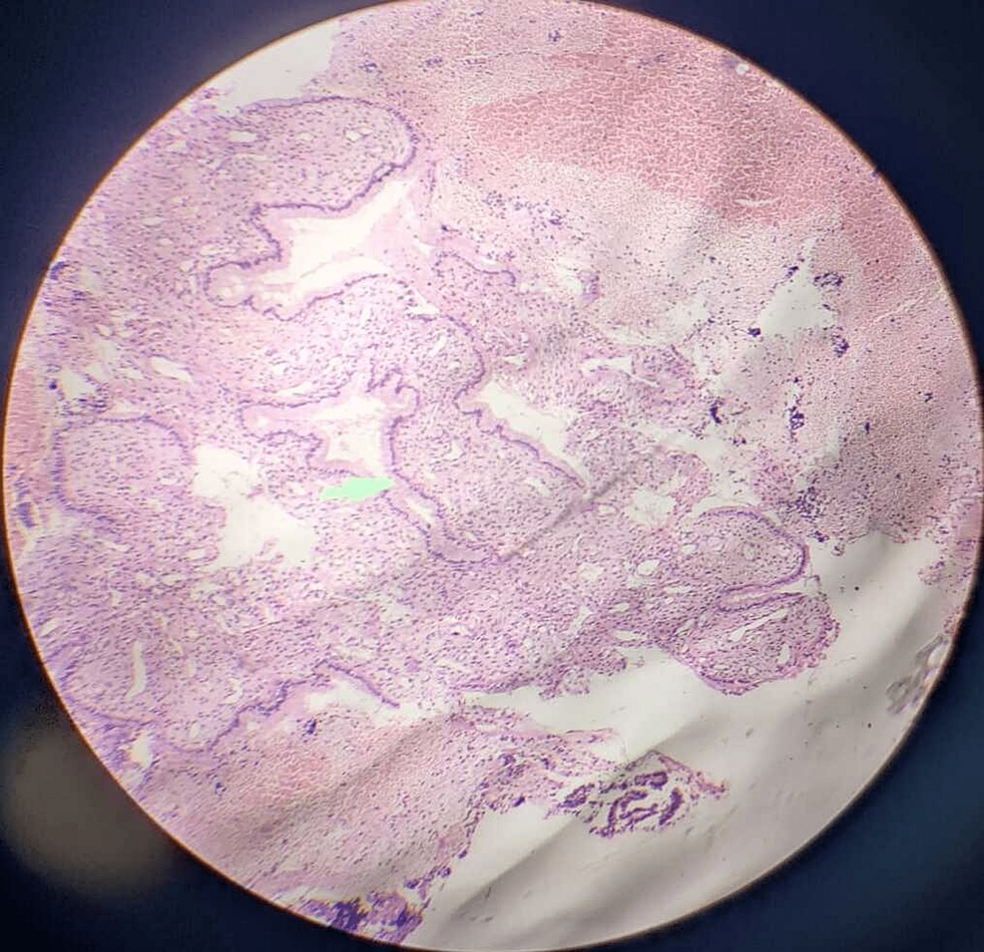
Histology of endometrial polyp (H and E stain) H and E = hematoxylin and eosin

**Figure 4:**
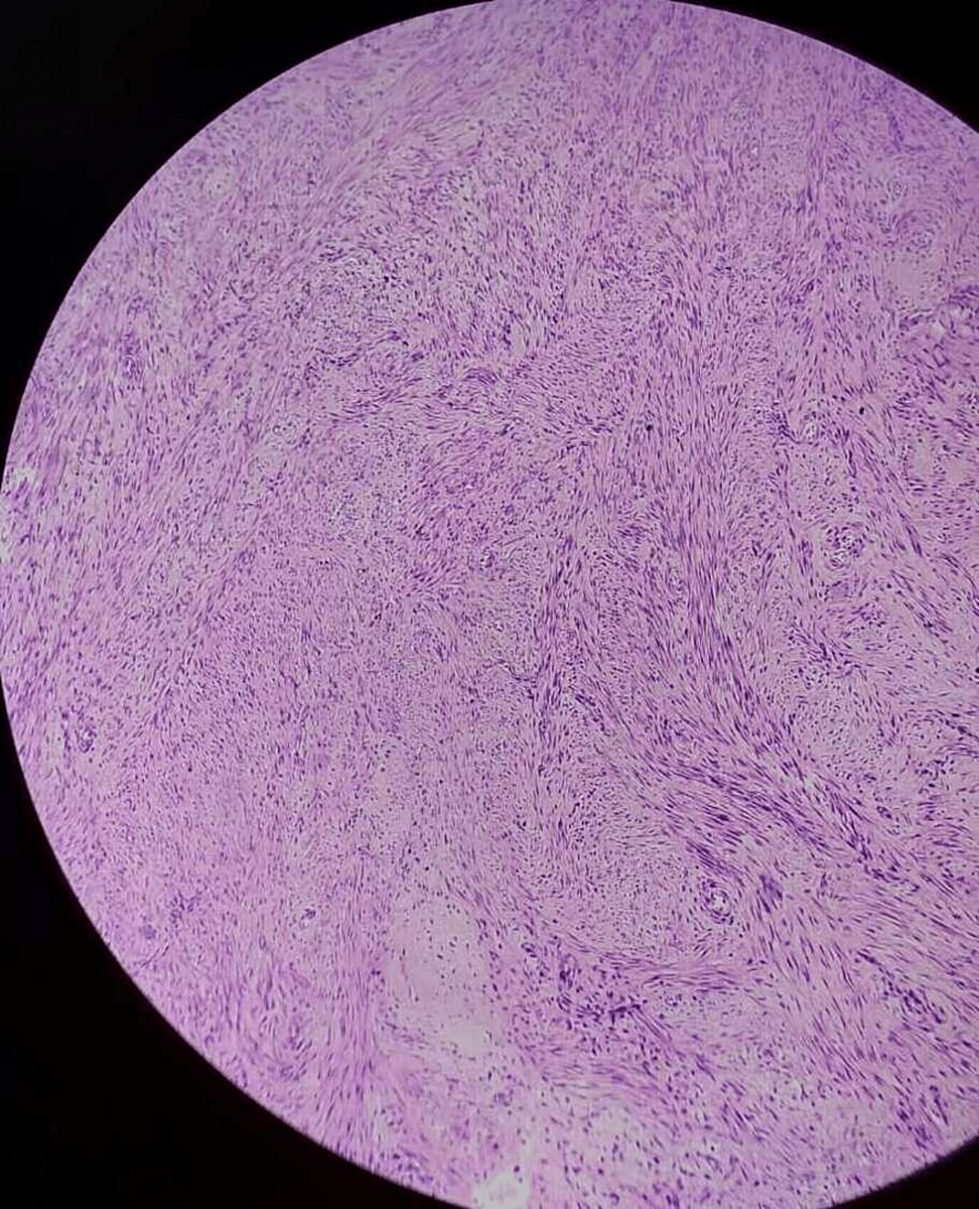
Leiomyoma showing whorl pattern and striations (H and E stain) H and E = hematoxylin and eosin

After a histological investigation, a diagnosis of dysfunctional uterine hemorrhage may merely be made by ruling out organic reasons. In DUB, three patterns are frequently observed. The foremost is "estrogen breakthrough bleeding," which happens when a "persistent follicle" produces estrogen continuously. The proliferative endometrium (Figure 2) swells to the point where it outgrows its own blood supply, resulting in breakthrough bleeding. The second is estrogen withdrawal hemorrhage, which occurs as a result of a failed follicle, in which two follicles produce subnormal estrogen levels. Both of these factors have been linked to anovulation [5-6]. The third is "ovulatory endometrium," which is caused by luteal phase and follicular phase abnormalities. The cyclical release of estrogen and progesterone from the ovaries regulates the typical cyclical physiological changes that occur in the endometrium of females during the reproductive period. The concentrations of receptors for these hormones differ as well. In particular, type I estrogen and progesterone hormones are expressed in hyperplasia and endometrial malignancies. The amount of these hormones in the body provides predictive information. They are also amenable to exogenous hormonal treatment. The key factor in the development of DUB is hormonal imbalance. The endometrium is examined histopathologically to better understand this imbalance. Pathological diseases such as chronic endometritis, endometrial polyps (Figure 3), and submucosal leiomyomas (Figure 4) can all produce abnormal bleeding [7].

The most prevalent reason for endometrial samples by endometrial biopsy or curettage is abnormal uterine bleeding. After medical explanations have been ruled out, endometrial biopsy or curettage is the gold standard procedure for evaluating AUB. AUB considers both organic and non-organic factors. Depending on the age group, distinct morphologic patterns and causes of AUB are observed. Endometrial biopsy or curettage histological assessment reveals a wide range of histomorphological patterns due to normal and pathological alterations such as exogenous hormone influences, infections, hyperplasia, and cancer. It also aids in the patient's overall care [8].

The morphological spectrum of endometrial pathology

Women of all ages have abnormal uterine bleeding without structural abnormalities. Dysfunctional uterine hemorrhage is the prevalent name for these instances. DUB is a non-organic illness with a hormone imbalance as the etiopathogenic substrate. Follicular maturation, ovulation, and corpus luteum production may be disrupted as a result of abnormalities along the hypothalamic-pituitary-ovarian axis, resulting in hormonal alterations. Abnormal uterine bleeding may result from these changes in typical hormonal cycles. The endometrium responds to estrogen stimulation by proliferation in the absence of ovulation and progesterone production. Endometrial growth without periodic removal causes the fragile endometrial tissue to burst. As seen in all of these circumstances, the bleeding is typically painless and erratic. Perimenopausal years are the ones with the highest prevalence of anovulatory cycles, and prolonged anovulation is associated with an unpredictable and erratic pattern of bleeding [9]. Studies have revealed that women with abnormal uterine bleeding account for 16.1% and 23%, respectively, of the study population that shows secretory endometrium [9,10].

Review findings

Due to a decrease in the number of ovarian follicles and their increased resistance to gonadotrophic stimulation, women's cycles shorten and often become intermittently anovulatory as they approach menopause. This causes a drop in estradiol levels due to a decrease in the number of follicles, which can no longer keep the normal endometrium growing. Endometrial assessment is used to determine malignancy or pre-malignancy in the endometrium, as well as to measure the endometrium's hormonal impacts. When a woman's bleeding pattern does not improve after a three-month course of medicinal therapy, endometrial histology should be evaluated [11]. Histopathological evaluation revealed proliferative endometrium in 45.7% and secretory in 30% of cases, as mentioned by Mahapatra et al. in their study [12]. In the study by Mahapatra et al., the most common presenting feature was menorrhagia (48.6%). Nayak et al., 1996 found menorrhagia in 49.1% of cases, similar to their study [12-13]. In their study, Zawar et al. (2005) reported 43% of cases with proliferative endometrium [14]. Doddamani et al. (2014) observed proliferative endometrium in 44.7% and secretory in 23.5% of cases [15].

According to Khan et al., out of 120, 102 patients had taken up for dilatation and curettage (D & C), and the endometrial tissues were sent for histopathological examination. The maximum numbers of cases were of endometrial hyperplasia (20.5%), followed by luteal phase insufficiency (15.6%) and secretory endometrium (13.7%). The least number of cases accounted for hormonal therapy changes. It was possible in 73 cases to compare the results of hormonal cytology with histopathological findings. Upon histopathological examination, hyperestrogenic states revealed an estrogenic smear on vaginal cytology. This was seen in 85.5% of endometrial hyperplasia cases and all cases of endometrial polyps, proliferative phase, and anovulatory cycles. However, only one case (12.5%) of endometritis had an estrogenic endometrial smear [16].

In their study, Abid et al. found that 66% (159/241) of participants had endometrial pathologies, compared to participants with a normal menstrual pattern. Among 82 cases of normal pattern endometrium, the secretory pattern was seen in 60% (49/82) of the participants, and the proliferative pattern in 40%. Hormonal imbalance pattern was the most prevalent pathology (41%, 65/159 cases) among 159 cases of endometrial pathologies. In 65 cases, the histological spectrum of the hormonal imbalance pattern was estrogen related in 80% (52/65) and progesterone related in 20% (13/65). The perimenopausal age group (54%, 35/64) had the highest prevalence of the hormonal imbalance pattern. This correlates with the fact that perimenopausal age is the transition from normal ovulation to anovulation [17].

In their study, Jetley et al. found that menorrhagia (46.4%) was the most frequent clinical manifestation, followed by metrorrhagia (20%), menometrorrhagia, polymenorrhea, and polymenorrhagia, among others. When the endometrium was examined, different histopathological patterns were found; the majority of the diagnoses were explained by functional causes. Secretory endometrium, seen in 71 cases (32.4%), was the most common. Proliferative endometrium was the second most typical diagnosis found in histopathology, occurring in 67 patients (30.5%). Endometrial hyperplasia was observed in 24 (10.9%) cases, with simple hyperplasia without atypia present in 19, complex hyperplasia without atypia present in four, and complex hyperplasia with atypia present in one. Other diagnoses that explained the remaining functional causes of atypical uterine bleeding included disordered proliferative endometrium in 15 cases (6.8%) and luteal phase defects in three cases (1.3%), according to research [18].

Sarwar et al. observed in their study that the most common complaint was polymenorrhagia 36% (18/50) followed by menorrhagia 30% (15/50). There were two cases of oligomenorrhoea (two percent) and five cases of post-menopausal bleeding (10%). Estrogen dominance pattern (42%, 21 cases), anovulatory endometrium (24%, 12 cases), chronic endometritis (two percent, one case), atrophic endometrium (two percent, one case), and pill effect endometrium (six percent, three cases) were the most common endometrial pathologies. According to their study, cases of abnormal uterine bleeding tend to cluster around the perimenopausal age. There was relative estrogen excess, termed estrogen dominance pattern (EDP) over progesterone, leading to specific changes such as clusters of thickened blood vessels, spindly stroma, weak or absent secretory changes in the glands, and stroma with or without polyp formation. We also noted a frequent anovulatory pattern in old age [19].

Chhatrasal et al. in their study observed that AUB was most prevalent in the perimenopausal age group. The most common presenting complaint was menorrhagia (45.5%). Endometrial hyperplasia was the most common histopathological finding and was seen in 25.3% of patients, with simple hyperplasia without atypia being the predominant pattern (22.6%), followed by secretory endometrium in 24.1% of patients, irregular ripening of endometrium in 19.5% of patients, and a proliferative phase pattern in 7.6% of patients. Malignancy was detected in 2.0% of cases, and endometrial carcinoma with 1.8% was the most common lesion. Histopathological evaluation of endometrial samples is, especially, indicated in women with AUB to rule out malignancy and preneoplasia. Among the patients with no organic pathology, normal physiological patterns with proliferative, secretory, and menstrual changes were observed. The most common endometrial pathology was endometrial hyperplasia. The rise in cases of irregular ripening of the endometrium is associated with abnormal hormonal effects [20].

Bhatta et al. conducted a study in which they reported that in AUB the most common endometrial histopathological finding was proliferative endometrium in 32 cases (26.23%), followed by simple hyperplasia without atypia in 22 cases (18.03%). Malignant lesions were more common in patients more than 40 years of age and comprised seven cases (5.74%). Atrophic endometrium was the most common finding [21].

In a study by Sharma et al., they discovered that 47.55% of perimenopausal women presenting with AUB are between the ages of 41 and 50, making them the most frequently affected age group. The most common histomorphological pattern in these patients was proliferative endometrium (38.8%), followed by secretory endometrium (16.3%), endometrial hyperplasia seen in 22 cases (12.0%), hormonal changes/pill endometrium was seen in six percent of the cases and least number cases were of endometrial carcinoma, forming 1.1% of the total cases taken in the study [22].

## Conclusions

Although hysterectomy is still the most popular option for abnormal uterine bleeding, healthcare practitioners must promote other techniques to ensure that women get the most benefits with the least amount of morbidity. In terms of histopathology, the majority of the cases with endometrial polyps are the most prevalent pathology in both reproductive and postmenopausal women. Relative estrogen excess over progesterone results in specific changes such as clusters of thickened blood vessels, spindly stroma, and weak or absent secretory changes in the glands and stroma with or without polyp formation. As a result, AUB care should be more customized and limited to preserve the uterus. If the biopsy is non-diagnostic, hormonal imbalance assessment in such cases may explain the AUB in postmenopausal women without other histopathological explanations.
